# 

**Published:** 1924-03

**Authors:** 


### HOSPITAL and HEALTH

REVIEW

*Established 1886* *No.* 1872. *Vol. LXXIII*

*New Series.* Vol. III. No. 30. *March,* 1924. Price Sixpence

CONTENTS.

| | PAGE |
|---|---|
| Houses and Slums | 65 |
| Exchequer Grants and the Public Health | 66 |
| Notes and Comments :— | |
| The Hospitals Association Conference—A Parliament of Health — The Ancient Scourge—The Invalid Ex-Service Man—"Red Cotton Night-Cap Country"—The Exchange of Medical Officers—A Little Question of Heraldry—Suggestion and the Tractor—Blunderbuss Pharmacy—The Doctor's Bill—Protective Eye-Glasses for Manual Workers—Innocent Mouthpieces—Cheap Rattlesnakes—The Widow's Claim—Tax-Free Bequests : a Solution — Set a Thief—— | 67 |
| Hospital Men of Mark | 71 |
| Public Health and Preventive Medicine | 72 |
| Co-ordination of Local Health Services | 73 |
| Blackburn and a Clean Milk Supply | 74 |
| Sheffield White and Sheffield Black | 75 |
| "The Beacon Light" | 76 |
| Health and Ventilation : Artificial Methods Condemned | 77 |
| The Care and Cure of Cripple Children | 78 |
| Health Week and Its Objects : A Story of Progress | 79 |
| Hospital Problems in Canada : Work of 1923 | 80 |
| Health of the World | 81 |
| Panel Fees : A Question of Ethics | 82 |
| Hospital Progress and Finance | 83 |
| Review of Books | 85 |
| A Hospital Pageant | 88 |
| Correspondence | 89 |
| A Cure for Leprosy at Last | 90 |
| Organising a Health Week : Prize of £100 | 91 |
| Echoes of the Month | 92 |
| Hospital and Health News | 93 |
| Points from Hospital Reports | 95 |
| Answers to Correspondents | 96 |
| Recent Appointments | 96 |

